# Genomic basis of the evolution of vertical bars in clownfishes

**DOI:** 10.1186/s12864-026-12570-9

**Published:** 2026-01-27

**Authors:** Lucy M. Fitzgerald, Thibault Latrille, Anna Marcionetti, Théo Gaboriau, Diego A. Hartasánchez, Nicolas Salamin

**Affiliations:** Department of Computational Biology, University of Lausanne, Génopode, Lausanne, 1015 Switzerland

**Keywords:** Pomacentridae, Selection, Patterning, Coloration, Ancestral reconstruction

## Abstract

Clownfish exhibit striking color patterns, characterized primarily by the presence of zero to three vertical white bars, along with three main colors: orange, white, and/or black. The common ancestor of clownfish likely possessed three vertical bars, with several instances of gains and losses occurring throughout clownfish evolutionary history over the past 10 million years. However, the evolutionary genomic mechanisms underlying the gain or loss of vertical bars remain unknown. In this study, we tested whether vertical bar transitions across the clownfish phylogeny were associated with changes in non-synonymous to synonymous substitution rates ($d_{\text {N}} / d_{\text {S}}$ values). Our analyses identified pigmentation-related genes that underwent changes in selective pressure, including *gch2*, *oca2*, and *vps11*, which are linked to melanophores, iridophores, and visual function. Additionally, *pmel*, a key melanogenesis gene, was found under positive selection, suggesting its role in shaping bar patterning. These results provide new insights into the genomic basis of coloration in clownfish, highlighting how selection and genetic variation influence phenotypic evolution.

**Significance statement** Clownfish are one of the most iconic groups of reef fish. Besides their mutualism with sea anemones, their conspicuous color patterns have fascinated evolutionary biologists and aquarium hobbyists alike. The number of vertical bars are variable between species and are thought to be used in species recognition, although little is known about how differences in their number of bars may have evolved. We have reconstructed the most likely evolution of number of vertical bars across the clownfish phylogeny finding both gain and loss events. We found that certain pigmentation-related genes underwent changes in selective pressures or positive selection alongside the gain or loss of vertical bars, providing evidence for selection driving the evolution of vertical bar patterns.

**Supplementary Information:**

The online version contains supplementary material available at 10.1186/s12864-026-12570-9.

## Background

Coloration in organisms can serve as a pivotal element in facilitating various ecological and evolutionary functions, such as signaling through inter- or intraspecific communication. Reef fish exhibit a wide range of color patterns, such as eyespots, vertical bars, and horizontal stripes, which can serve multiple purposes, including communication and predator-prey interactions [1]. For instance, colorful stripes in cleaner gobies (*Elacatinus* species) have been shown to facilitate communication in a cleaning mutualistic relationship, where the blue stripes of cleaner gobies serve to advertise their role to client fish, promoting cleaner-client interactions and deterring predators. In particular, yellow-striped gobies, while less conspicuous to predators, engage in cleaner-client interactions less frequently than their blue-striped counterparts, highlighting the evolution of coloration from predator deterrence to a more specialized signaling function [2]. Similarly, in butterflyfishes (*Chaetodon* species), stripes and eyespots have traditionally been linked to predator defense, such as misdirecting attacks or deterring predators. However, a comparative analysis suggests that these markings are evolutionarily dynamic and may also be shaped by ecological factors such as habitat type, sociality, and dietary complexity, rather than serving exclusively as anti-predator adaptations [3]. Thus, reef fish coloration plays a critical role not only in survival through predator avoidance but also in social behaviors, including territorial interactions and species recognition. The evolution of these patterns is likely driven by a complex interplay of selective pressures, highlighting the diverse functions of coloration in marine environments.

Within the reef fish family of *Pomacentridae*, the clownfish clade *Amphiprioninae* is a unique group of 28 species that form an obligate relationship with up to 10 species of sea anemones (genera *Entacmaea, Heteractis, Radianthus*, and *Stichodactyla*), and this association was suggested to have triggered their adaptive radiation [4, 5]. Clownfish have a relatively simple pattern of three main colors (orange, white and/or black) and zero to three vertical white bars. Some species, mostly from the skunk species complex, have a horizontal stripe across the top of their head and body [6]. The clownfish clade is a monophyletic group evolving from a shared common ancestor, and within the group, there are species pairs with different color patterns that can be leveraged to study the evolution of color patterning.

In the literature, several hypotheses have been proposed about the role of color patterns in clownfish [7]. One is species recognition, which serves as a form of communication between individuals with different numbers of white bars. In juvenile *Amphiprion ocellaris*, individuals were more likely to attack other individuals with different numbers of vertical bars than those with the same number of bars [8]. Additionally, observations of interspecies cohabitation are rare, but when it occurs, they never have the same number of vertical bars. For example, individuals from *A. clarkii* (two or three bars) can be found with *A. perideraion* individuals (one bar). Similarly, individuals of *A. biaculeatus* (three bars) can coexist with *A. melanopus* individuals (one bar) [9]. Another suggested role of color patterns is protection from predators, as the contrasting colors and white bars help hide the fish silhouette within the sea anemone tentacles [10]. Finally, a function of aposematic signaling, in which clownfish use their striking colors to advertise the toxicity of their host sea anemone has also been proposed [11]. However, these hypotheses are not conclusive to the function of coloration and bar patterning in clownfish. Recently, it was found that the coloration of clownfish is significantly linked to their host sea anemone [5]. Individuals hosted by *Radianthus* sea anemones tend to be more orange/yellow, those hosted by *Entacmaea* are darker orange/red, and those that are generalists (inhabiting more than three anemone species) are darker with contrasting vertical bars.

Understanding the function of clownfish color patterns provides important context for investigating how these traits evolved. Ancestral state reconstruction can help clarify whether certain patterns were historically linked to ecological pressures such as species recognition, predator avoidance, or aposematic signaling. One study [11] used a maximum likelihood estimation to infer the ancestral vertical bar patterns, treating the number of vertical bars as a continuous trait. They showed that the ancestral clownfish had, on average, 2.73 vertical bars, and evolutionary transitions most likely led to a bar loss rather than a gain. The loss of vertical bars was not exclusive to the clownfish clade but occurred several times across different parts of the phylogenetic tree of the *Pomacentridae* family. In contrast, a second study [10] used stochastic character mapping, which is a Bayesian approach that incorporates uncertainty in character evolution as a discrete trait. They showed that the common ancestor of clownfish likely had three vertical bars approximately 12 million years ago. The color patterns seen in mature individuals of extant species could thus result from the loss of their vertical bars caudal-rostrally, during development. In certain species, vertical bars are lost sequentially from their tail to their head during development. This pattern is not universal; in many species the number of bars remains constant from juvenile to adult stages, indicating that ontogenetic bar loss is restricted to specific clades. The authors found nine species exhibiting bar loss during ontogeny from five distinct clades [10]. Both of these earlier studies relied on a phylogeny based on seven nuclear markers [12], which has since been updated using whole genome-data for all 28 species [5]. Using this updated phylogeny from Gaboriau et al. [5], Mitchell et al. [13] reconstructed ancestral states and identified three clades in which 10 species lose bars during ontogeny. While juvenile bar counts provide valuable insight into trait evolution, comprehensive data are currently available for only a subset of species, limiting the feasibility of a complete phylogenetic analysis.

To confirm these evolutionary patterns, it is essential to investigate the molecular mechanisms underlying color production and pattern formation. In teleost fish, there are three main types of pigment cells (also known as chromatophores), namely, melanophores, xanthophores, and iridophores, as well as less common types such as leucophores, erythrophores, and cyanophores [14]. Several studies have linked these chromatophores to specific genes in fish [15–17]. These studies suggest that pigmentation is influenced by a combination of genetic factors, with some genes having more significant effects than others. Gene expression can also play a fundamental role in shaping phenotypic plasticity, particularly in relation to clownfish color patterns although only few studies have been done. For example, in *A. percula*, thyroid hormones (TH) regulate the expression of *duox*, a gene involved in pigment cell development. Increased *duox* expression leads to faster white bar formation, a process influenced by their anemone host species. Juveniles form white bars faster when associated with *Stichodactyla* compared to *Radianthus* anemones [16].

While gene expression governs immediate phenotypic responses, protein-coding sequences are subject to evolutionary pressures that shape long-term trait evolution. Genes associated with pigmentation and visual perception may experience different selective regimes, depending on ecological or behavioral constraints. For example, genes involved in visual perception could be under positive selection in species where visual acuity plays a crucial role in predator detection or mate choice. Similarly, pigmentation genes may evolve under different selective pressures depending on the ecological or social context. In *Pomacentridae*, a study examining selection in six opsin genes within the family found all but one visual opsin gene under positive selection [18]. Additionally, they found cases of divergent, parallel, convergent, and reversed evolution across the damselfish family, highlighting the importance of visual acuity in the group. Given the link between TH-mediated gene expression and pigmentation, genes regulating white bar formation in clownfish may be also evolving under specific selective pressures. A study identified 128 pigmentation-related genes in clownfish, categorized by pigment cell type [16], providing candidate genes for further evolutionary analysis.

Pigmentation changes across species can sometimes be explained by mutations in a single gene with large effects (e.g., *MC1R* [19] or *ASIP* [20]). However, patterning traits such as the presence or absence of vertical bars in clownfish likely involve multiple developmental pathways. They may be influenced by different genes contributing to bar formation, maintenance, or loss. We therefore hypothesize that distinct genes under selection are related to the gain or loss of white vertical bars in clownfishes. Genes essential for maintaining bars are likely under stronger purifying selection, whereas those associated with bar loss may exhibit relaxed selection. Conversely, if vertical bars provide an adaptive advantage such as species recognition, genes involved in their formation may experience directional selection. By examining changes in selective pressure across pigmentation genes and mapping these changes onto the phylogenetic tree, we aim to identify signatures of directional selection or changes in selection pressure associated with vertical bar evolution and clownfish coloration. By integrating phylogenetic and genomic approaches, this study provides insights into the molecular mechanisms underlying vertical bar evolution. These findings will contribute to a broader understanding of how selection shapes phenotypic diversity and adaptation in marine organisms.

## Methods

### Data

All coding sequences used in this study were obtained from the clownfish genome assemblies published by Gaboriau et al. [5], which include the 28 species. From these sequences, we focused on two datasets. The first dataset consisted of 128 pigmentation genes previously identified by Salis et al. [16] and categorized by their main pigment cell type (melanophores, xanthophores, iridophores; see Supplementary Table S5). Orthologs of these 128 genes were retrieved from all 28 species using reciprocal best BLAST to *A. frenatus* [21], resulting in 79 genes included in the “Color Genes” dataset (Supplementary Table 1). The second dataset, referred to as the “All Genes” dataset, includes 18,390 protein-coding genes shared across the 28 species (and contains the 79 “Color Genes”). Adult vertical bar number was recorded for each species (zero to three) following Salis et al. [10] (Supplementary Table 2). Polymorphic traits in *A. clarkii* and *A. polymnus* were not included, but the assigned number of bars from Salis et al. [10] was retained.

### Ancestral state reconstruction and branch categorization

**Fig. 1:**
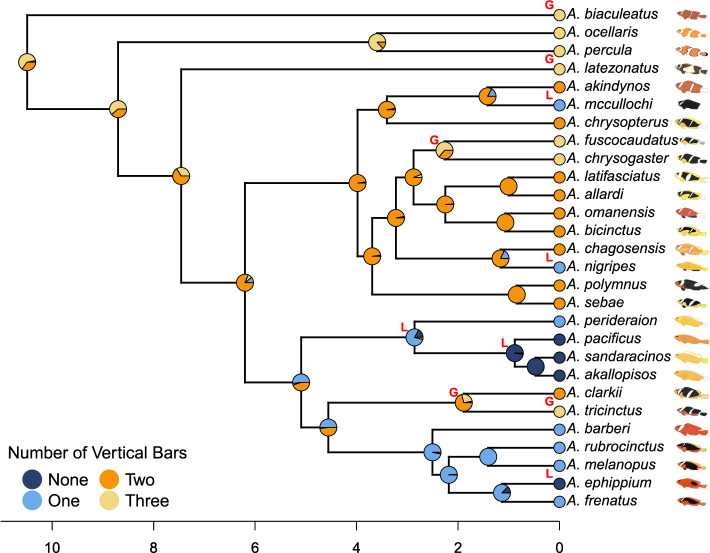
The summary stochastic map of the reconstructed number of vertical bars with the four states: None, One, Two, and Three. Pies at each node represent the marginal probability of each ancestral number of bars. “G” labels to the right of the node refer to branches categorized as Gain and “L” as Loss

We reconstructed the ancestral state of the number of vertical bars as adults using the R package*CorHMM* (v2.8) [22]. We tested different transition matrices corresponding to six evolutionary models: (1) all rates differ, (2) symmetric, (3) equal rates, (4) biological all rates differ, (5) biological symmetric, and (6) biological equal rates (Supplementary Table 3). Biological models 4-6 assumed equal transition rates between any consecutive state (e.g., one to none, or two to three) and did not allow transitions between non-consecutive states (e.g., one to three). The best model was selected based on the Akaike Information Criterion (AIC) [23]. We then used the R package *phytools* (v2.1-1) [24] to generate 100 stochastic maps under the best model. The summary of these 100 stochastic maps is shown in Fig. 1. For each stochastic map, we assigned a number of bars to every internal node of the phylogeny using our ancestral state reconstruction. For each branch, we then recorded whether the number of bars increased (gain), decreased (loss), or remained the same between the parent and child nodes. The frequency of each type of transition across all stochastic maps was used as a measure of how often that branch experienced each state (Supplementary Figure 1, Filtered output). Weight differences for each branch were calculated by subtracting the loss weight from the gain weight.

### Detection of differential selective pressure associated with the evolution of bar number

To test for an association between the gain or loss of bars and changes in selective pressures, we relied on the $d_{\text {N}} / d_{\text {S}}$ ratio, which is the non-synonymous substitution rate ($d_{\text {N}}$), which is compared to the synonymous substitutions ($d_{\text {S}}$), or $\omega$ [25, 26]. The values of $\omega$ signify different forms of selection: $\omega < 1$ indicates negative (purifying) selection, preserving protein function; $\omega > 1$ suggests positive selection, potentially driving functional divergence; and $\omega = 1$ indicates neutral evolution [27]. Relaxed selection, where purifying constraints are reduced, can contribute to greater phenotypic variation even with $\omega < 1$ [28].

In this framework, methods such as *BayesCode* [29] or RELAX [30] detect shifts in the strength of natural selection by comparing whether selection has been relaxed or intensified across each branch of a phylogenetic tree. The change in selective pressures acting on genes can be modeled as changing along branches of the phylogenetic tree  [31]. This modeling provides a more nuanced understanding of selection, and the Bayesian framework allows for the detection of changes in selective pressures associated with other traits [29, 32]. Although relaxed selection indicates a weakening of purifying constraints, changes in selection can also manifest as positive selection when advantageous mutations are favored. CodeML (PAML) [33] seeks $d_{\text {N}} / d_{\text {S}}$ values greater than 1 for some specific sites of the protein-coding genes as an indication of positive selection.

We estimated $\omega$ changes along the clownfish phylogenetic tree using a Bayesian approach implemented in the *BayesCode* software [29] using the following commands:

$$\begin{aligned} & \texttt{nodeomega}\ \, \text {-}\texttt{a}\ \, \texttt{my}\_\mathtt {alignment.phy}\ \, \text {-}\texttt{t}\ \, \texttt{my}\_\mathtt {tree.newick}\ \, \text {-}\texttt{u}\ \, \texttt{2000}\ \, \texttt{my}\_\texttt{genename}\\ & \texttt{readnodeomega}\ \, \text {-}\text {-}\texttt{every}\ \, \texttt{1}\ \, \text {-}\text {-}\texttt{until}\ \, \texttt{50000}\ \, \text {-}\text {-}\texttt{burnin}\ \, \texttt{5000}\ \, \text {-}\text {-}\texttt{newick}\ \, \texttt{my}\_\texttt{genename} \end{aligned}$$

In *BayesCode*, the prior of $\omega$ along the tree is simulated as a Brownian process, and its value changes at each node of the tree, or, in other words, it is increasing or decreasing along each branch of the tree. The topology of the tree was fixed to correspond to the clownfish species tree of Gaboriau et al. [5], while node ages and changes in mutation rate along the tree were jointly estimated by *BayesCode* [29]. We then obtained posterior estimates of $\Delta \omega$, which corresponds to the changes in $\omega$ along each branch due to the Brownian motion modeling used in *BayesCode*. Altogether, we obtained one posterior $\Delta \omega$ per branch and gene. In our analyses, a positive $\Delta \omega$ implies an increased $\omega$ value on the focal branch, indicating some relaxation of purifying selection or increase of positive selection, which both indicate that more changes were occurring at the molecular level. In contrast, a negative $\Delta \omega$ could suggest a tightening of selection that limits changes at the molecular level because the estimated $\omega$ is smaller in the most recent branch. For each gene, to test whether changes in selective pressure along branches of the tree were associated with the loss or gain of bars, we used a linear model to predict $\Delta \omega$ as a function of bar changes (loss, same, gain). We obtained a single $\Delta \omega$ value per branch. The ancestral number of bars can differ across different stochastic maps (each stochastic map is a single scenario of bar gain/loss leading to the current observations), so each branch could be classified as a gain, loss, or no change in a variable proportion across the different stochastic maps. To account for this uncertainty, we performed a weighted regression using the proportion of stochastic maps in which each type of bar change occurred (loss, gain, same) as the independent variable (Supplementary Figure 1). The significance of the weighted regression was assessed as a *p-*value for each gene. Across the genes tested, we corrected for multiple testing using the *p.adjust* function and the Benjamini-Hochberg method implemented in the R package *stats* (v4.2.3) [34]. Significantly enriched Gene Ontology (GO) terms (http://geneontology.org/) of the significant genes were identified using Fisher’s exact test in the topGO package [35].

### Testing for positive selection

To investigate the presence of genes under positive selection linked with the gain or loss of vertical bars, we used the branch-site model H1 (model = 2, fix omega = estimate) compared to a neutral model H0 (model = 2, fix omega =1) as implemented in CodeML (PAML, v.4.9) [33] across the “All Genes” dataset. We categorized branches (gain or loss) from the ancestral state reconstruction as foreground branches in the branch-site model. The model was run separately for the two types of transitions (i.e., gain or loss; Supplementary Figure 2). We followed the recommendations from PAML, in which CodeML was run three times for the null and the alternative hypothesis with the initial branch length ignored to allow for three randomized runs of the optimization process to increase the chances of reaching the optimum. The highest likelihood for each test run was saved for the likelihood ratio test (df=1). We extracted both the Bayes empirical Bayes (BEB) [36] and Naive empirical Bayes (NEB) [37] values from the CodeML output. Then we corrected the *p-*values across all genes for multiple testing using the *p.adjust* function and the Benjamini-Hochberg method implemented in the R package *stats* (v4.2.3) [34]. Lastly, significantly enriched GO terms (http://geneontology.org/) of the significant genes were identified using Fisher’s exact test in the topGO package [35].

### Detecting expression of selected genes in clownfish skin

Since the list of “Color Genes" were not clownfish specific, we wanted to check if the significant genes from our analysis were expressed in clownfish. We downloaded transcriptomic data of orange and white skin from *A. ocellaris* (Bioproject PRJNA482578) from Salis et al. [14] and then ran HiSat2 (v2.2.1)[38] to map the reads to the *A. ocellaris* reference genome [39]. Raw gene counts were normalized to Transcripts Per Million (TPM) accounting for gene length and sequencing depth. For a gene to be considered “expressed" in a particular skin type (orange or white), its TPM value was required to be greater than 1 in at least two out of three biological replicates for that skin type.

## Results

### Repeated gain and loss of vertical bars across the clownfish phylogeny

The number of vertical bars at each node of the phylogenetic tree [5] was inferred using ancestral state reconstruction. The best model was selected based on the lowest Akaike Information Criterion (AIC) value and corresponded to model 6 (AIC = 59.4), the biological equal rates model. We summarized the 100 replicates of stochastic mapping by recording the number of vertical bars inferred at each node and estimating the probability of each state (Fig. 1). The root node of the clownfish phylogenetic tree was inferred to have three vertical bars with probability = 0.62 (two bars: probability = 0.35; one bar: probability = 0.03). This number then evolved through gains and losses of bars within the main clownfish clades. Throughout the phylogenetic tree, we have evidence for repeated cases of loss and gain of vertical bars. We identified two internal branches (ancestors to *A. tricinctus* and *A. fuscocaudautus*) and three terminal branches (*A. biaculeatus*, *A. latezonatus*, *A. tricinctus*) as gain. In addition, two internal branches (ancestors to *A. perideraion* and *A. pacificus*) and three terminal branches (*A. mccullochi*, *A. nigripes*, and *A. ephippium*) were identified as loss. Most of the nodes are categorized as “same,” meaning that the number of vertical bars in the parent and child branches was identical (Supplementary Figure 1). The two losses of vertical bars at the base of the skunk complex might reflect the same selective pressure acting sequentially, first on the caudal and mid bars, followed by the head bar. Similarly, the consecutive gains leading to *A. tricinctus* could also result from similar selective pressures occurring in two independent evolutionary events.

### Genes associated with gain or loss of vertical bars

**Fig. 2:**
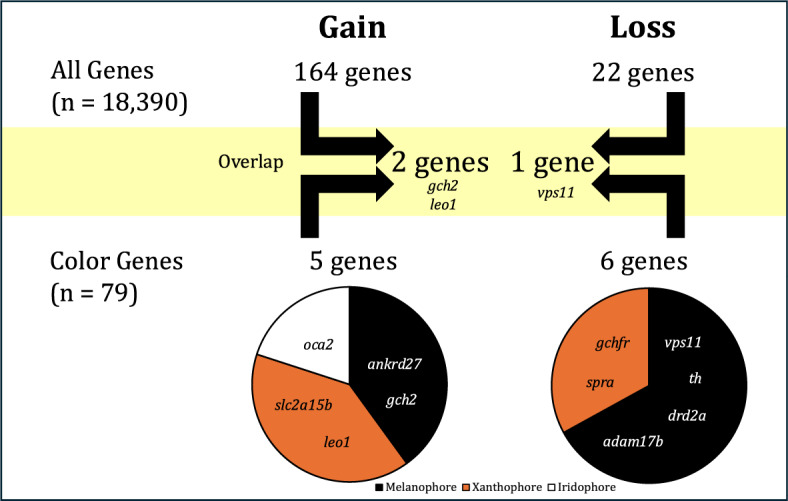
Summary of the number and name of significant color genes found in each dataset (All Genes and Color Genes) for gain and loss and their pigment cell type under differential selection

**Fig. 3:**
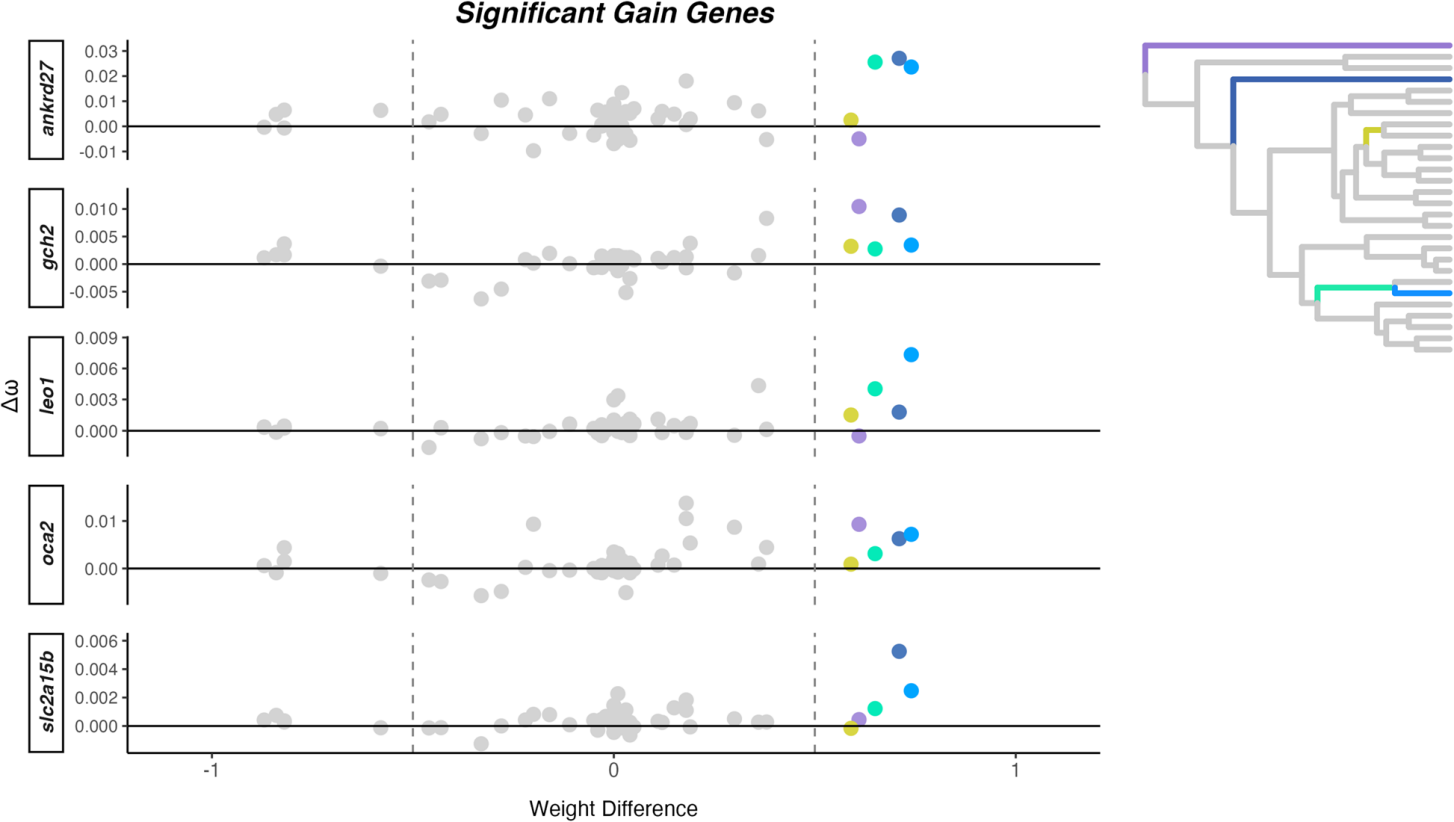
$\Delta \omega$ and weight difference for five color genes significantly associated with vertical bar gain. Each dot represents a branch on the phylogenetic tree (n = 54), with colored dots representing branches that are categorized as gain, colored in the phylogenetic tree (upper right corner; a larger version is in Supplementary Figure 2)

**Fig. 4:**
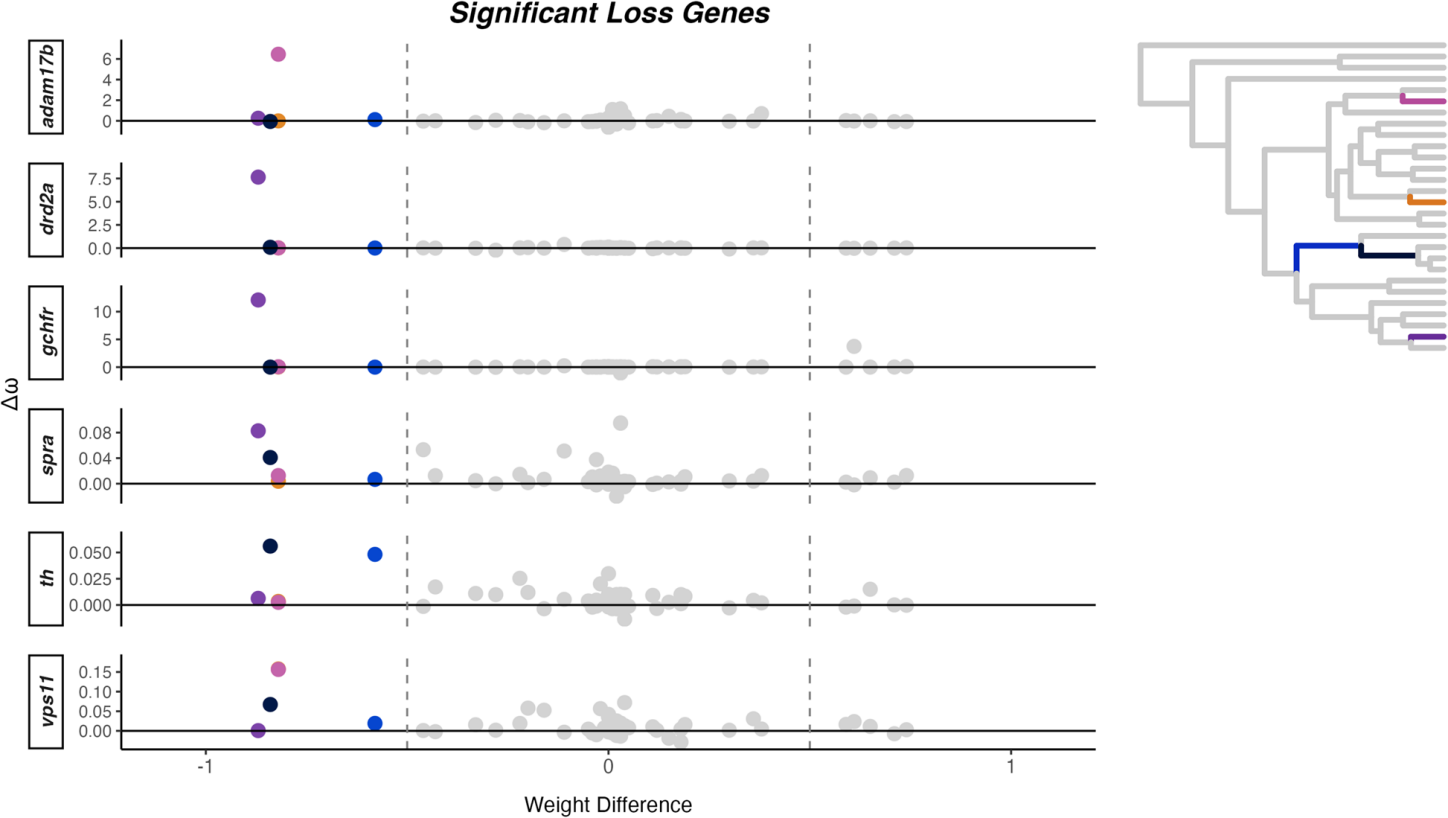
$\Delta \omega$ and weight difference for six color genes significantly associated with vertical bar loss. Each dot represents a branch on the phylogenetic tree (n = 54), with colored dots representing branches that are categorized as loss, colored in the phylogenetic tree (upper right corner; a larger version is in Supplementary Figure 2)

Out of the 18,390 orthologous genes used in our study, we found 164 genes showing patterns of significant shift in selection pressure, $\Delta \omega$, associated with vertical bar gain, while 22 genes were associated with vertical bar loss (Supplementary Table 4). We also obtained average $\omega$ values for gain or loss for each significant gene (Supplementary Table 5). There were no GO terms associated with coloration or vision in the significant genes associated to vertical bar gain (n=164) or loss (n=22) (Supplementary Table 6). Within the significant genes associated with bar gain, we found two coloration genes, *gch2* and *leo1*, and for bar loss, one coloration gene, *vps11* (Fig. 2). Testing only within the “Color Genes” dataset, we found five genes with $\Delta \omega$ linked to vertical bar gain, and six genes with $\Delta \omega$ linked to vertical bar loss (Fig. 2). Genes associated with the gain of bars represent all three categories of pigment cells categorized by Salis et al. [16], whereas only melanophores and iridophores are represented among genes linked to bar loss. Within the five genes related to vertical bar gain (*ankrd27*, $\omega$ = 0.40; *gch2*, $\omega$ = 0.16; *leo1*, $\omega$ = 0.09; *oca2*, $\omega$ = 0.05; and *slc2a15b*, $\omega$ = 0.06; see Supplementary Table 5), we found different branches driving $\Delta \omega$ in the significant coloration genes (Fig. 3). Similarly, within the six coloration genes significantly related to vertical bar loss (*adam17b*, $\omega$ = 0.20; *drd2a*, $\omega$ = 0.11; *gchfr*, $\omega$ = 0.06; *spra*, $\omega$ = 0.22; *th*, $\omega$ = 0.28; and *vps11*, $\omega$ = 0.20; see Supplementary Table 5), we also found different branches driving $\Delta \omega$ in the significant coloration genes (Fig. 4).

### Positively selected genes related to vertical bar gain

**Fig. 5:**
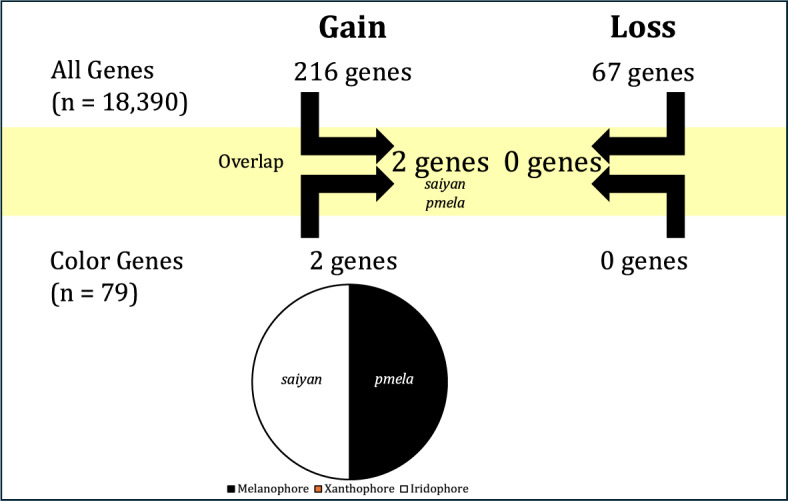
Summary of the number and name of significant color genes found in each dataset (All Genes and Color Genes) for gain and loss and their pigment cell type under positive selection

Across the entire dataset (18,390 genes), we found 216 significant genes under positive selection associated with vertical bar gain, and 67 genes under positive selection with vertical bar loss (Supplementary Table 4). We also calculated the average foreground $\omega$ values for each significant gene (Supplementary Table 5). There were no GO terms associated with coloration or vision in the significant genes related to vertical bar gain or loss (Supplementary Table 6). Among the 216 genes related to vertical bar gain, two coloration genes were found, *saiyan (si:ch211-256m1.8)* and *pmela*. Although both Bayes empirical Bayes (BEB) and Naive empirical Bayes (NEB) methods were used, the amino acid sites under selection were quite variable and did not converge. Only the NEB sites were found to be significant in the two genes of interest, even though CodeML suggests that BEB results should be prioritized. However, among the 67 genes, there were no coloration genes found for vertical bar loss (Fig. 5). Interestingly, the two coloration genes found under positive selection for vertical bar gain were related to iridophores and melanophores, respectively.

### Expression of color genes in clownfish skin

We also checked the 477 genes that were significant across all analyses (Supplementary Table 4) to see if any of these genes were expressed in the white and orange skin of clownfish, *A. ocellaris*, from Salis et al. [14]. We found that overall 266 genes were expressed, with 258 genes expressed in both white and orange skin, seven genes expressed in white skin only, and one gene was expressed in orange skin only (Supplementary Table 7). Of these, only genes previously implicated in pigmentation or coloration are discussed below. Significant color genes (average orange TPM; average white TPM) *adam17b* (19.6; 22.1), *ankrd27* (2.5; 7.1), *gch2* (4.3; 3.2), *gchfr* (31.0; 35.5), *leo1* (28.6; 34.7), *spra* (10.4; 14.1), and *vps11* (20.0; 21.6) were expressed in both, *slc2a15b* (0; 1.2), *pmela* (0; 1.0), and *saiyan (si:ch211-256m1.8)* (0; 4.7) were expressed in white skin only. Genes *drd2a*, *oca2*, and *th* were not expressed in these samples.

### Overlapping significant genes

We found only three significant genes (*traf4a*, *adgrd1,* and an unknown gene) that overlapped between the *BayesCode* gain analysis (n = 164) and CodeML gain analysis (n = 216), and none of them are linked to coloration. However, *traf4a* was expressed in both white and orange skin (8.7; 8.6) of *A. ocellaris*. No genes were found between *BayesCode* loss analysis and CodeML loss analysis.

## Discussion

In this study, we investigated the genomic substrate underlying the evolutionary gain or loss of vertical bars in clownfish. Given previous ancestral state reconstruction in clownfish [10, 11], we expected that transitions in vertical bar patterns would be non-random and play a significant role in the evolutionary history of the group. Specifically, shifts in vertical bar presence or absence could be associated with ecological factors such as habitat complexity, social signaling, or changes in predation pressure. Based on this, we hypothesized that coloration genes involved in bar formation and maintenance would be under selection, either due to functional constraints maintaining the pattern or adaptive pressures favoring its modification. Our findings support this hypothesis, revealing that a handful of pigmentation-related genes exhibit signatures of positive selection and changes in selection pressure.

Our results of the ancestral state reconstruction revealed a high probability that the root of the clownfish clade had three vertical bars in line with previous studies [10]. Vertical bar gain and loss appear to have occurred multiple times throughout clownfish evolution, with equal numbers of transitions in both directions. This is particularly interesting since prior studies [10, 11] have primarily focused on vertical bar loss rather than gain, whereas our results suggest that both processes are integral to clownfish coloration evolution. Different lineages may experience selection on this trait at different life stages; however, juvenile bar count data are currently available for only a subset of species, so our ancestral state reconstruction focuses on adult counts, which are consistently documented across all 28 species. The repeated loss and gain of vertical bars across independent lineages coincides with shifts in ecological specialization, particularly in reproductive host associations. Recent work has shown that clownfish specializing in different sea anemone hosts exhibit convergent color patterns, with generalists typically retaining three white bars, whereas specialists tend to have a reduced number of bars or altered pigmentation [5]. This pattern suggests that transitions in vertical bars could be linked to host-driven selection pressures, where specialists experience relaxed selection on disruptive coloration or adaptive shifts favoring different visual signals. Our findings suggest this idea, hinting that vertical bar transitions have played a dynamic role in the evolutionary history of clownfish, potentially shaped by their ecological niches and host associations.

Since vertical bars transitions occurred multiple times in the clownfish evolution, we investigated if these changes were associated with shifts in selection. Using a Bayesian framework, we tested for signs of changes in selective pressure on pigmentation-related genes and genes that could be related to pigmentation. More significant genes were recovered within the “All Genes" dataset compared to the “Color Genes" dataset due to increased statistical power despite correcting for multiple testing. Our analysis identified several coloration genes with elevated rates of evolution associated with vertical bar gain (*ankrd27*, *gch2*, *leo1*, *oca2*, and *slc2a15b*). The $\Delta \omega$ values for these genes are very low (0.006–0.03), in contrast to some bar loss-related genes, which exhibited $\Delta \omega$ values up to 7.5. While low $\Delta \omega$ values most likely indicate a slight relaxation of purifying selection, large $\Delta \omega$ values reflect much stronger changes in selective pressure, potentially implying positive selection. Nevertheless, $\Delta \omega$ values are not necessarily correlated with the extent of the phenotypic change associated with it.

Moreover, these genes are linked to all three pigment cell types found in clownfish: melanophores, xanthophores, and iridophores. While Salis et al. [16] provided a candidate list of coloration genes, we found specific examples that link how these genes directly affect color patterns in clownfish. It has been shown that mutations in *gch2* (expressed in both white and orange skin of *A. ocellaris*) alters the xanthophore pigmentation in zebrafish larvae, but in adults, normal pigmentation is restored [40]. Additionally, *slc2a15b* (expressed in white skin only in *A. ocellaris*) plays a role in xanthophore and leucophore differentiation in teleost fish [41]. CRISPR mutations of *oca2* lead to albinism in cavefish by disrupting the melanin pathway [42]. While this gene was not expressed in the skin of *A. ocellaris* [14], it was recently found to be downregulated during bar loss in juvenile *A. frenatus* [13]. The absolute $\omega$ values for bar gain-related genes are elevated compared to genes under strong purifying selection, but generally remain below the threshold expectation under strong positive selection (absolute values of $\omega > 1$). The pattern of increased $\omega$ along branches ($\Delta \omega > 0$) is consistent with a relaxation of purifying selection, but it is not proof of strong positive selection (i.e., $\omega \ge 1$), Relaxation of purifying selection may have allowed greater genetic variation to accumulate and potentially facilitated the recurrent evolution of vertical bars in different lineages. Conversely, genes associated with vertical bar loss showed highly variable $\Delta \omega$ values, with three genes (*gchfr*, *drd2a*, and *adam17b*) exhibiting very large $\Delta \omega$ on a single branch (well exceeding 1), consistent with either strong positive selection or a relaxation of purifying selection. Additionally, *adam17b*, was found to be upregulated during bar loss of juvenile *A. frenatus* (pers. comm. Laurie Mitchell). These genes were only found to be related to melanophores and xanthophores, which control black and orange pigmentation, respectively. One gene of interest, *vps11*, which is expressed in both white and orange skin in *A. ocellaris*, has been linked to melanophore development [43] and visual function in zebrafish [44]. Given the importance of vision in species recognition within *Pomacentridae* [18, 45], the association between vision-related genes and bar loss may reflect adaptations in visual signaling.

The two genes found under positive selection were exclusively associated with vertical bar gain, with no significant findings for vertical bar loss. This distinction suggests that while increased rate of evolution plays a role in facilitating bar loss, directional selection may be maintaining or facilitating the re-emergence of bars in certain lineages. Although they are both significant, the amino acid sites under selection were quite variable and not convergent. None of the coloration genes under increased rate of evolution were found under positive selection, which is consistent with the idea that such changes in selection allows for accumulation of neutral mutations, whereas positive selection favors functional modifications. Given that the genes under positive selection were exclusively linked to vertical bar gain, we examined their potential functional roles in pigmentation. Interestingly, *saiyan* (*si:ch211-256m1.8*) was described by Salis and colleagues as significantly expressed in white skin cells of *A. ocellaris* and *A. frenatus* with no previous associated function [14] and was now found to be downregulated during bar loss in juvenile *A. frenatus* [13]. The other gene, *pmel* (expressed in *A. ocellaris* white skin), is essential for melanogenesis [46]. Additionally, *pmel* has also been implicated in pattern transition from blotches to stripes in snakes [47].

Our analyses revealed limited overlap in significant genes. Only three genes (*traf4a*, *adgrd1,* and an unknown gene) were significant in both the *BayesCode* gain analysis (n = 164) and CodeML gain analysis (n = 216), with none being directly linked to coloration. Notably, *traf4a* was expressed in both white and orange skin of *A. ocellaris*, despite not being annotated as a coloration gene. In other species, *traf4a* has been implicated in immune function and cold-water adaptation [48], suggesting that genes with non-color functions can still be expressed in skin and affect pigmentation, highlighting the importance of functional studies beyond previously identified color genes.

Additionally, six genes overlapped between the CodeML gain and loss analyses, but these genes were not functionally annotated. These minimal overlaps highlight key methodological differences: *BayesCode* identifies changes in selective pressure along a branch across the whole gene, indicating reduced evolutionary constraint, whereas CodeML detects positive selection, indicating functional divergence at specific sites of the gene. Given these differences, a lack of overlap is expected, as they do not capture the same signal of selection acting on sites and genes.

Although we identified many additional significant genes associated with vertical bar gain and loss beyond known coloration genes, no significant GO terms related to color, pigmentation, or vision were detected. However, this does not mean these genes are unrelated to coloration; rather, their roles may simply not be annotated as such in current databases.

## Conclusions

Overall, our findings contribute to a broader understanding of how selection shapes phenotypic diversity and adaptation in marine organisms. We identify several coloration genes under selection, which have been shown to be functionally related to color in other species, highlighting their potential role in clownfish coloration. The next step in validating these candidate genes is functional testing via CRISPR/Cas9, which has been successfully applied in some clownfish species but remains limited in scope [49]. Additionally, full transcriptomes of the 28 species could be useful for better understanding the role of certain genes involved in coloration that may differ between species but obtaining such a dataset is challenging. Future studies integrating gene expression analyses with functional assays will be crucial for determining the mechanistic basis of vertical bar evolution in clownfish.

## Supplementary Information

Supplementary Material 1.

## Data Availability

All code is available on Github: https://github.com/phylolab/clownfishBarEvolution.git.
